# *YoyoMut*: Interactive Exploration of SARS-CoV-2 Mutation Fixation and Reversion Through Time

**DOI:** 10.3390/life16050776

**Published:** 2026-05-06

**Authors:** Jana Penic, Tommaso Alfonsi, Giovanni Chillemi, Ingrid Guarnetti Prandi, Fabrizio Maggi, Anna Bernasconi, Daniele Focosi

**Affiliations:** 1 Department of Electronics, Information, and Bioengineering, Politecnico Di Milano, 20133 Milano, Italy; jana.penic@mail.polimi.it (J.P.); tommaso.alfonsi@polimi.it (T.A.); 2 Bioinformatics Research Unit in Infectious Diseases, National Institute for Infectious Diseases Lazzaro Spallanzani-IRCCS, 00149 Rome, Italy; giovanni.chillemi@inmi.it; 3 Department of Experimental Medicine, University of Rome “Tor Vergata”, Via Montpellier 1, 00133 Rome, Italy; 4 Independent Researcher, Italy; ingrid.prandi@gmail.com; 5 Laboratory of Virology, National Institute for Infectious Diseases Lazzaro Spallanzani-IRCCS, 00149 Rome, Italy; fabrizio.maggi@inmi.it; 6 North-Western Tuscany Blood Bank, Pisa University Hospital, 56126 Pisa, Italy; daniele.focosi@gmail.com

**Keywords:** SARS-CoV-2, viral evolution, reversion, fixation, cyclic mutation, vaccines, dashboard, 3D-protein structures

## Abstract

Reversion of amino acid mutations in structural proteins is common in viral evolution. SARS-CoV-2 provides an unprecedented opportunity for ecological studies, thanks to the abundance of available whole genome sequences. *YoyoMut* allows regular scanning of open SARS-CoV-2 data, reporting on all cyclic and reverting mutations within all proteins (including Spike), with fine-grained trend visualization distinguishing non-mutated from mutated positions (either fixated or cyclically reversed). In the whole CoVSpectrum database, order of 100 reverting and 50 fixated mutations were identified on Spike. Classification is determined using alternative algorithms (based on threshold or slope inversion); finally, a 3D-protein structure allows us to identify spatial clustering of adjacent mutated positions. Systematic, automated monitoring of these behaviors aids immunologists and structuralists in their manual curation. By generating informative reports, our tool supports daily activities that have practical implications for vaccine and therapeutic anti-Spike monoclonal antibody design: prioritizing analysis of cyclic mutation and reversion models could help avoid the recent failures in their development and inform future strategies.

## 1. Introduction

Since the outbreak of a worldwide pandemic in 2019, the large amount of SARS-CoV-2 sequencing data has allowed for a wide analysis and monitoring of genomes’ evolution and mutations.

Specifically, several amino acid residues located in relevant regions of SARS-CoV-2 have undergone cyclic mutation and reversion during the pandemic. Such mutations should be taken into consideration and tracked as they can change the effectiveness of monoclonal antibodies and vaccines [1], as well as confer the ability of faster replication or increased resistance to the existing immune reaction of the host [2]. A particular focus is on the Spike protein, representing the main target of COVID-19 vaccines and neutralizing antibodies, subject to selective pressures [3,4,5].

In this manuscript, we describe the development, use, and results of the *YoyoMut* tool, which analyzes and classifies patterns of mutation behavior observed in SARS-CoV-2. *YoyoMut* allows flexible and customizable exploration of cyclic mutation and reversion, including (but not limited to) the Spike protein. Moreover, visualization of mutation locations on 3D structures supports analysis for vaccine and therapeutic mAb manufacturing.

*Related work.* Recent work has attempted predicting SARS-CoV-2 evolution with generative-AI based approaches [6,7], but the vast landscape of bioinformatics online tools (reviewed in 2023 by Cheng et al. [8] and in 2024 by Vello et al. [9]) does not comprehend yet a lightweight tool such as *YoyoMut* for plain analysis of mutation trends. While only CoV-Spectrum [10] allows this kind of analysis mutation per mutation, it does not run automatic reporting specifically classifying mutations based on their temporal trend. With respect to tools we built previously, also focused on single-mutations analysis [11,12], *YoyoMut* supplies completely parametric modules for classification of trends and downloadable reports scanning the whole available open data.

*Manuscript organization.* In this manuscript, we overview materials and methods, propose our resulting web application, briefly evaluate its parameter space and manually validate a subset of interesting results, concluding with a discussion on prospective use of the platform for supporting genomic surveillance of SARS-CoV-2 and other viruses.

## 2. Materials and Methods

### 2.1. Characterization of Mutated Positions

**Figure 1:**
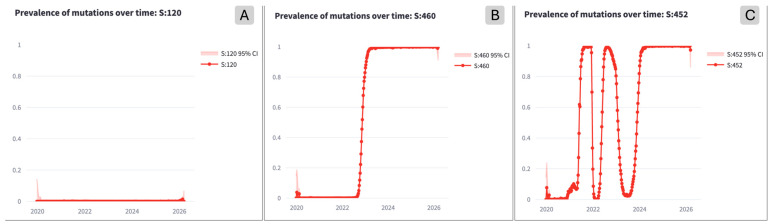
Example of mutation prevalence profiles of unmutated residue at Spike:120 (**A**), fixated residue at Spike:460 (**B**), and *yo-yo* residue at Spike:452 (**C**).

Mutations in SARS-CoV-2 are detected with respect to the reference Wuhan1 genome sequence [13]. When analyzing the temporal evolution of residue mutations, three patterns can be recognized: *unmutated*, *fixated*, or *reverting* (i.e., *yo-yo*) [14,15]. The difference is based on the concept of *prevalence*; the prevalence of a mutation *m* in a span of time *t* is defined as the ratio of sequences exhibiting *m* in *t* over the total of sequences collected during *t*. It spans the [0, 1] interval, where 0 indicates that no sequence with *m* appeared in *t*, while 1 indicates that all sequences had that mutation in the same period of time. For each location where a mutation at the amino acid level (with respect to the wild type) has been observed in the dataset, we capture prevalence weekly, starting in 2020. The choice for prevalence’s weekly binning is driven by typical countries’ data submission processes, which happen on a weekly basis; this choice also meets minimal requirements to generate robust significance and interpretation. We adhere to the choice already defended in previous works that study prevalence trends [16,17,18]. Figure 1 exemplifies the differences:

- *Unmutated* residues are those that have the same amino acid as the wild-type virus (i.e., original, naturally occurring strain of a virus, as it exists in nature before significant mutations occur).
- *Fixated* residues are those where the mutation of an amino acid residue happened and has not reverted to the original amino acid since.
- *Yo-yo* residues appear for a period of time, possibly changing characteristics of the viral products, but later revert to the wild-type amino acid or to a new mutation. Note that, in the following of the manuscript, when mentioning *yo-yo* or *reverting* mutations we refer precisely to this operational definition based on prevalence dynamics rather than a direct description of molecular evolutionary processes. These residues can be a challenge for the development of vaccines and therapeutic monoclonal antibody manufacturing. The reverting mutations happen as an adaptation to increased immune pressure and reduced fitness. After the environmental pressure on the virus changes, the mutations can revert [15].

**Figure 2:**
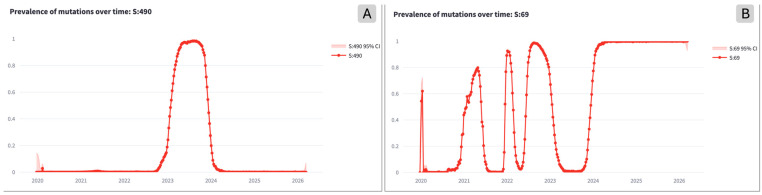
Example of mutation prevalence profiles of *yo-yo* mutations with a single hill at Spike:490 (**A**) or multiple hills and final saturated prevalence at Spike:69 (**B**).

The most intriguing pattern is the last one; our analysis follows from [15], where several *yo-yo* mutations were recognized manually. Interestingly (see Figure 2), we observe that a landscape of different profiles can be characterized as *yo-yo*: some have only one hill, others more hills, others show a final 100% prevalence (i.e., half a hill).

**Figure 3:**
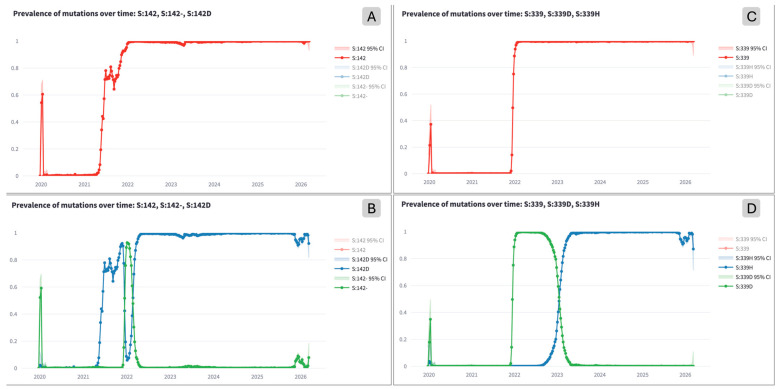
Prevalence trend of (**A**) the generally-mutated position Spike:142, (**B**) the corresponding specific mutations, i.e., the substitution Spike:142D and the deletion Spike:142-, (**C**) the generally-mutated position Spike:339, and (**D**) the corresponding specific mutations, i.e., the substitutions Spike:339H and Spike:339D.

Single mutations are analyzed as both: (i) *generally-mutated positions*, where any amino acid other than the original wild-type counts as a mutation, and (ii) *specific mutations*, where the specific alternative amino acid is indicated in the mutation name. By comparing generally-mutated positions and specific mutations, we found that mutations on the same residue—but resulting in different amino acids—can show different prevalence trends, clearly reflecting a preference of the virus toward a specific residue. Computationally, generally-mutated position patterns correspond to the sum of values obtained by all the possible alternative residues. As an example, Figure 3 includes two noteworthy mutations; panel A shows the trend of the generally-mutated residue at position 142 classified as fixated; panel B has the specific mutations S:142D and S:142- (‘-’ marks deletion), both classified as *yo-yo* mutations; panel C shows the fixated position S:339; panel D shows S:339D as a *yo-yo* mutation and S:339H as a fixated mutation.

### 2.2. Data Pipeline

#### 2.2.1. Data Acquisition

**Figure 4:**
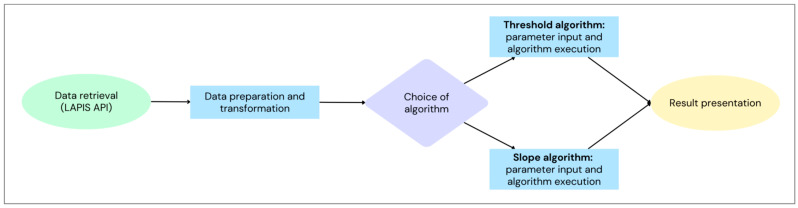
Data retrieval and processing pipeline of the *YoyoMut* framework.

The main source of data is CoV-Spectrum [10], which contains publicly available GenBank data [19]. Data is retrieved weekly through the LAPIS API that allows filtering and aggregation [20]. The pipeline of the system is shown in Figure 4: we perform data retrieval, followed by data preparation and transformation. Users may select two alternative algorithms, which are run on the transformed data according to user-set parameters, producing per-mutation results in tabular form and protein-structure visualization.

During data retrieval, we make an API GET request to the https://lapis.cov-spectrum.org/open/v2/ path (accessed on 7 April 2026), requiring the sample/aminoAcidMutations endpoint. This returns a JSON file whose entries represent residue mutations, with the following fields: *mutation* (mutation name, e.g., “S:L452R”), *count* (number of sequences containing the mutation, e.g., 4106994), *sequenceName* (name of the gene it is located on, e.g., “S”), *mutationFrom* (“L”), *mutationTo* (“R”), *position* (452), *coverage* (e.g., 8427985), and *proportion* (e.g., 0.487304379397923). To improve significance, we filter data based on the *count* information; we retain mutations that appeared in at least 1000 sequences. For each of them, we collect per-day counts.

#### 2.2.2. Data Transformation and Mutation Classification

For each considered mutation, we prepare data aggregated and averaged over 7-day periods or over a predetermined number of sequences. The data then contains: start/end dates of the aggregation period; number of sequences with the mutation; total number of sequences collected; ratio of sequences with mutation out of the total number of sequences; and upper/lower boundaries of the 95% confidence interval. Two different algorithms were developed for the classification of residue mutations.

*Threshold algorithm.* Data is grouped and averaged over 7 days. The algorithm classifies residues based on whether their prevalence surpassed a certain threshold. Parameters are the *threshold* and the *minimal duration* in days to remain above the selected threshold. Based on this selection, the algorithm classifies mutations as *fixated* if the mutation prevalence rose above the selected threshold and remained above it, and as *yo-yo* if the prevalence of a mutation rose above the threshold, stayed above for at least the minimum duration specified, and then dropped below the threshold.

*Slope algorithm.* It classifies residues based on recognizing the positive and/or negative slope values of the relative frequencies of mutations. First, the number of data points used for slope calculation is chosen. The slopes are calculated on sliding windows of the data obtained by smoothing over a predetermined number of sequences (currently, *n* = 5000), ordered by collection date. The calculated slope values are then analyzed chronologically. If the slope is positive, which indicates the growth of prevalence, the mutation is marked as *started*. If the slope stays non-negative, it is classified as a *fixated* mutation. If the slope value becomes negative, it is classified as a *yo-yo* mutation (i.e., with at least one hill).

#### 2.2.3. Different Proteins Support

Residues of interest are visualized on the 3D structures of their protein. We include the structural proteins E, M, N, S (where N is divided into the C and N terminals); the Open Reading Frames ORF3a, ORF7a, ORF8, ORF9b; and the non-structural proteins NSP2, NSP3, NSP5, NSP9, NSP10 (part of ORF1a), and NSP13, NSP15 (part of ORF1b). The model of 3D structure of the S glycoprotein was built by using SWISS MODEL [21] by alignment of the reference sequence to the cryo-EM structure (PDB ID: 6VYB) [22], according to the process described in [23]. Similarly, for the other proteins, we retrieved the viral protein composition from UniProt and selected from PDB the best structures, considering both resolution and number of missing residues. The visualization is created using 3Dmol.js [24], a JavaScript library that transforms data from PDB files to a 3D rendering of a structure.

#### 2.2.4. Architecture and Automatization

*YoyoMut* is a client-server architecture-based application, implemented with Python v3.15 and the open-source framework Streamlit [25]; the whole architecture has been packaged as a Docker container. To ensure continuous updates of the application content, automated scheduling was implemented using jobs of the Unix-based scheduler Cron. At the time of writing, the data retrieval and processing pipeline is scheduled to run every Sunday at 03:00 CET on our servers. On startup, the application loads prepared data from the file-based system into memory. After the initial loading into memory, data remains static and is used as input for the algorithms.

## 3. Results

The web interface, freely available at http://gmql.eu/yoyomut/ (accessed on 7 April 2026) enables choosing the parameters, running the identification algorithms, and showing the results of the analyses. It includes a home page, two pages for algorithm analyses, a page for presenting the results of the classified residue on 3D protein models, and a page where the results can be downloaded as a report.

### 3.1. Running the Algorithms

By inputting the necessary parameters and running the algorithm, users get a summarized result of the number of mutations classified as unmutated, fixated, or *yo-yo*, separated into generally-mutated positions and specific mutations.

**Figure 5:**
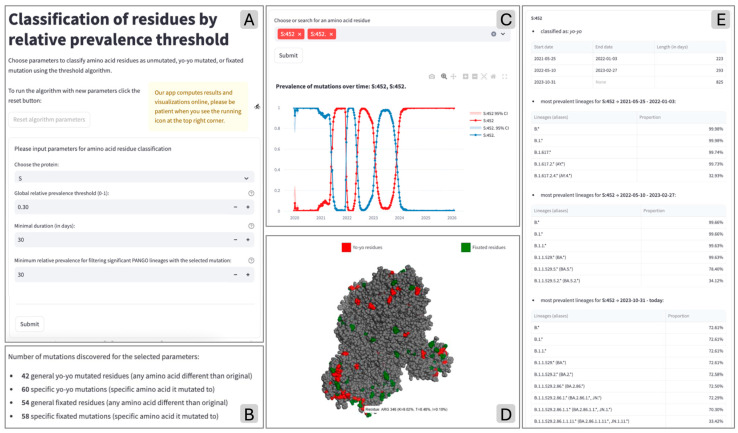
Overview of the main functionalities available in the tab for running the threshold algorithm. Users can set the parameters, submit the analysis (**A**), and retrieve a list of residue counts by type (**B**); then, they can specify positions and mutations, visualizing their temporal trends (**C**) and their positions on the protein (**D**). Finally, for each mutation, users can inspect their representation in groups of lineages (**E**).

Figure 5A shows the input form; Figure 5B shows the summary of the results obtained by running the threshold-based algorithm. Below the displayed counts, a section for building other plot and table visualizations is provided. Figure 5C shows the prevalence time series plotted for the example selection mutation S:452R; Figure 5D displays its interactive 3D protein structure, where users can observe if mutated residues tend to cluster within specific areas of proteins. Finally, a summary table is built for each of the selected mutations (see Figure 5E); this includes the specific time periods when the mutation was active (start/end dates and the length of the active mutation period). For each active period, the lineage information of the sequences is fetched and grouped by families (parental lineage contains its own counts, but also the sum of all of its child lineages, according to Pangolin [26]). In this specific example, we see that residue S:452 has created a first hill during which it mostly appeared in AY.* lineages, a second hill where it appeared in BA.5* lineages. Finally, it has become a fixated residue, with major prevalence in the JN.1* lineages.

### 3.2. 3D Model Visualization

A dedicated page of *YoyoMut* enables visualization of results on a 3D model of SARS-CoV-2 protein structures. After the parameters are chosen and the algorithms are run, the mutated residues (general mutations) can be seen on the protein structure model. Colors and styles of the visualization can be customized for both unmutated/fixated/*yo-yo* categories and for relevant domains in the structures. In the Spike protein, we consider the N-terminal domain (spanning the 27-292 coordinates), N2R (293-330), Receptor-binding domain (331-541), Antigenic supersite (14-20 + 140-158 + 245-264). The model can be rotated, zoomed in, zoomed out, or focused on a residue. By hovering over a residue, information about the residue name, the original amino acid, and a list of observed mutations is shown. Figure 5E shows the final result of the protein model visualization, where the label of residue 346 is shown, as it appears when hovering on the specific position of the 3D structure. The label contains information about the amino acid found in the wild-type, in this case arginine (ARG), and the prevalence of amino acids found in mutated sequences. On this residue, the most common mutation was to lysine (K), and it was found in 9.02% of sequences. The next mutation was to threonine (T), found in 8.48% of sequences, and lastly, 0.10% of sequences had a mutation to isoleucine (I).

### 3.3. Generating Reports

General reports on the results of the mutation classification can be generated. After choosing the algorithm and its parameters, two reports are generated, respectively for *yo-yo* and *fixated* mutations. Each page details the trends of one mutation (including both general and specific mutations). Future work will apply systematic analysis to assess whether there are tendencies of *yo-yo* mutations sites to cluster within particular functional regions.

### 3.4. Parameter Exploration and Qualitative Validation

The optimal choice of parameters is left to expert users through the interactive Web interface. However, here we assessed a variety of different combinations to understand the optimal perimeter of functioning of the tool when used on the CoV-Spectrum SARS-CoV-2 dataset. Both described algorithms have been tested using different parameter combinations. Experiments were run on specific mutations (rather than on generally-mutated positions) so as to consider the most fine-grained level available.

#### 3.4.1. Threshold Algorithm Results

We experimented with runs using a threshold in the [0.10, 0.70] threshold range and [30, 100] duration days range. Empirically, we observed an increase in sensitivity (in detecting hills) with decreasing parameter values, as detailed below.

Keeping the threshold fixed to 0.3, the algorithm produced comparable outputs for durations of 30, 50, and 70 days, whereas the outputs for 10 and 100 days deviated noticeably. If a mutation is considered active after only 10 days of prevalence exceeding the threshold, the algorithm classifies more *yo-yo* mutations and fewer fixated mutations. Instead, having a minimal duration of 100 days, more recognized mutations are classified as fixated rather than *yo-yo*. In other words, the sensitivity of the algorithm to classifying mutations as *yo-yo* increases as the minimal duration parameter decreases.

We then experimented by keeping the minimum duration fixed to 30 days and varying the threshold. As expected, the higher the threshold, the fewer mutations are observed. Mutations peaking at low frequencies are not common, as they would otherwise cause the lower thresholds to recognize much larger numbers of mutations than the higher thresholds.

Setting the parameter combination to (0.3, 30), we counted the number of *yo-yo* mutations by date of appearance, observing that the first peak was at the end of 2020 (coinciding with the emergence of multiple variants around the world, including Alpha, Eta, Kappa, Iota), with follow ups in May 2021, December 2021 (rise of the first Omicron variant and infection cases worldwide), and January 2023. Looking at the distribution of the hills’ length (i.e., active mutation periods), we observed that most have a duration between 40 and 240 days.

#### 3.4.2. Slope Algorithm Results

We experimented with runs using a progressive number of slope points in the [2, 25] range. We confirmed that fewer points used to calculate the slope make our algorithm more sensitive to recognizing *yo-yo* mutations. Because of noise in data fluctuations, using only two data points to compute the slope was not the ideal setting. Using 5 or more points led to more consistent results, where the number of *yo-yo* mutations decreases as the slope points increase, while the number of fixated mutations only slightly increases. The slope algorithm also revealed an increase in hill detection sensitivity with the decrease of slope points (input parameter). With 5/7 points used to calculate the slope, this algorithm is much more sensitive to data fluctuations compared to the threshold algorithm, whilst the results for parameter values 15 and 25 align more closely with the outcomes of the threshold algorithm.

Keeping the slope points fixed to seven, spikes of *yo-yo* mutations appeared in October 2020, March/April 2021, November 2021, and November 2022, typically 2 or 3 months in advance with respect to our results with the threshold algorithm.

The lengths of the hills recognized by the slope algorithm are longer in comparison to the threshold algorithm findings, with most instances spanning lengths [27, 327] days. This difference can be explained by the underlying mechanics of the algorithms; less if the increase is steep, more if the prevalence increases over a longer period of time. One such example is shown by Spike:446, where the slope algorithm recognizes a first hills lasting 149 days, while the same is recorded as 76 days for the threshold algorithm.

#### 3.4.3. Manual Validation

To supplement the previous analyses, we performed a sample validation of the two classification algorithms, based on manual flagging of fixated and *yo-yo* mutations in the Receptor-binding Domain (respectively inside and outside the Receptor-binding Motif, located at 437-508 positions of the Spike protein). Manual classifications are taken from the paper [15] where the review was done in October 2024.

We report our findings on Table 1. After the region and the position, we report the manual results; in the following columns, we report the current classification performed by *YoyoMut* with default parameters: Threshold Algorithm (0.30 global relative prevalence threshold, 30 minimal duration in days); Slope Algorithm (5 timepoints).

We note that the Threshold Algorithm is perfectly aligned with the manual flagging. The Slope Algorithm, instead, reveals more sensitive in cases where there are more fluctuations, recognizing as *yo-yo* also cases with very slight hills (i.e., 375, 405, 408, 417 outside of the RBM and 440, 477, 478, 484, 486, and 505 inside the RBM).

## 4. Discussion

*YoyoMut* stands as a data analysis tool with several practical features that may help the everyday work of immunologists. We overview possible fields impacted by using such an automatized approach.

*Monoclonal Antibody Development.* The development of monoclonal antibodies (mAb) as a treatment for COVID-19 is one of the treatments that would be of critical help to immunocompromised patients [27]. Most importantly, anti-Spike monoclonal antibodies are being researched as possible therapies. Because the target of the treatment is typically the Spike protein, mutations that happen in the gene coding region for Spike can change the effectiveness of the antibodies, as has happened with the emergence of the variant named JN.1 [27]. Mutations of the Spike protein coding gene can cause structural changes of the protein, thus creating possibilities of making developed mAbs ineffective. In conjunction with our tool *ConvMut* [28] (which studies the convergence of mutations along phylogenenies), *YoyoMut* can support the analysis of the temporal trends of mutations of interest, such as mutations that are specific for a new variant. Notably, *YoyoMut* identifies real-time if those mutations already had *yo-yo* behavior in the past or if they are fixated mutations, unlikely to change again.

*Spatial Relationships in Protein Structures.* Due to the tertiary and quaternary structure of the protein, the folding of the polypeptide chain and the assembly of multiple polypeptide chains into a protein, the spatial proximity of two residues does not follow linear rules. *YoyoMut* offers a practical 3D spatial model visualizer, where several SARS-CoV-2 proteins can be inspected, with highlighted locations of mutations. Some mutations, at first, might seem unrelated to each other, but their spatial proximity can better inform how these can significantly affect the shape and functionality of the protein.

*Variant Surveillance.* When a new variant is assigned a name and possibly is flagged as “variant of interest” or “variant under monitoring”, it is described as a combination of mutations [18], in line with what Outbreak.info [29] and Cov-Spectrum [10] do. These mutations can then be viewed using *YoyoMut*, supporting researchers to get structured and organized information about the mutations (trend visualizations, historical behavior, and classification).

One of the current Variants under Monitoring (as of 23 February 2026) is the recombinant lineage XFG (according to the Pango lineage nomenclature [30]). XFG samples were first documented on 27 January 2025; the lineage name was assigned only a few months later (25 June 2025). It is genetically defined using the older lineage JN.1, which has acquired S:T22N, S:S31P, S:K182R, S:R190S, S:R346T, S:K444R, S:V445R, S:F456L, S:N487D, S:Q493E, and S:T572I. This variant is of particular interest for its antigenic and virological characteristics [31,32].

**Figure 6:**
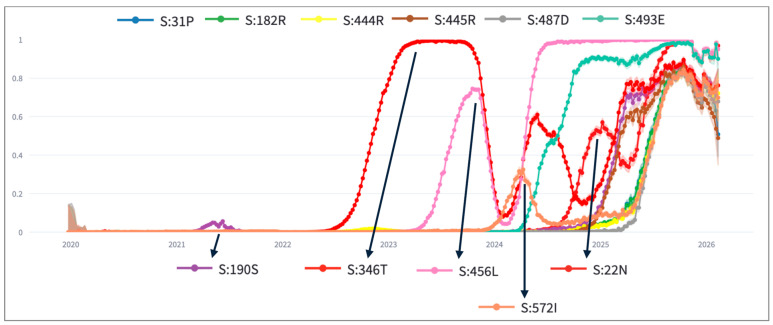
Prevalence over time of mutations characterising lineage XFG from JN.1.

By using *YoyoMut* (threshold = 0.3, minimum days = 30), we can easily plot the trend of all such mutations (see Figure 6). Out of 11 mutations that define the XFG variant in comparison to JN.1, only two are classified as *yo-yo* (S:346T and S:456L), even though more mutations have obvious *hills*; specifically, S:22N is flagged as *fixated* because, even though the mutation prevalence passes the threshold of 0.3, it does not drop under the threshold; S:572I is flagged as *fixated* because, even though it has a *hill* in early 2024, the prevalence remains above the threshold for less than 30 days (this would be instead be flagged as *yo-yo* using the slope algorithm with 5 time points). The slope algorithm would also flag as *yo-yo* S:190S, due to the small (but long) hill in early 2021.

*YoyoMut’s* approach is very general and parametric, so it could be easily extended to analysing temporal trends of mutations for other viral species, such as those already supported in the GenSpectrum (partner project of CoV-Spectrum) [33] for open data-reliant long-lasting genomic surveillance support.

On a more general perspective, *YoyoMut* was devised within our long-running contribution to SARS-CoV-2 data-driven surveillance, using both static analysis [18,34] and online-system monitoring [11,12,35,36,37]. *YoyoMut* provides an essential, scalable, and automated layer to the global genomic surveillance infrastructure, ultimately supporting faster and more informed public health responses worldwide, as a follow-up of the “data-driven Genomic Computing” and “SENSIBLE” projects vision [38,39].

## Figures and Tables

**Table 1:** Sample validation of classification algorithms comparing manual flagging [15] with *YoyoMut* algorithms results. Green color is used for matching values.

RBD Position	Residue Position	Manual val. (24 October)	Threshold Algorithm	Slope Algorithm
inside RBM	440	fixated	fixated	***yo-yo***
	445	fixated	fixated	fixated
	455	fixated	fixated	fixated
	460	fixated	fixated	fixated
	477	fixated	fixated	***yo-yo***
	478	fixated	fixated	***yo-yo***
	481	fixated	fixated	fixated
	483	fixated	fixated	fixated
	484	fixated	fixated	***yo-yo***
	486	fixated	fixated	***yo-yo***
	498	fixated	fixated	fixated
	505	fixated	fixated	***yo-yo***
outside RBM	332	fixated	fixated	fixated
	339	fixated	fixated	fixated
	371	fixated	fixated	fixated
	373	fixated	fixated	fixated
	375	fixated	fixated	***yo-yo***
	376	fixated	fixated	fixated
	403	fixated	fixated	fixated
	405	fixated	fixated	***yo-yo***
	408	fixated	fixated	***yo-yo***
	417	fixated	fixated	***yo-yo***
inside RBM	444	*yo-yo*	*yo-yo*	*yo-yo*
	446	*yo-yo*	*yo-yo*	*yo-yo*
	452	*yo-yo*	*yo-yo*	*yo-yo*
	456	*yo-yo*	*yo-yo*	*yo-yo*
	490	*yo-yo*	*yo-yo*	*yo-yo*
	493	*yo-yo*	*yo-yo*	*yo-yo*
	496	*yo-yo*	*yo-yo*	*yo-yo*
	501	*yo-yo*	*yo-yo*	*yo-yo*
outside RBM	346	*yo-yo*	*yo-yo*	*yo-yo*
	368	*yo-yo*	*yo-yo*	*yo-yo*

Note that, due to more update sequence data (and therefore counts), the two algorithms were able to recognize additional residues. The Threshold Algorithm also recognized residues 356, 450 and 487 as fixated. The Slope Algorithm recognized many new fixated resides (336 outside the RBM and 450, 487, 499, 500, 502, 504 inside of the RBM). Also *yo-yo* residues increased their representation; these include the ten cases indicated in bold in the last column of Table 1, with the addition of 475 and 521 inside the RBM (previously flagged as unmutated).

## Data Availability

All data used for this project is openly available through the CoV-Spectrum https://cov-spectrum.org/ (accessed on 7 April 2026) and the LAPIS API https://github.com/GenSpectrum/LAPIS (accessed on 7 April 2026). The implementation of *YoyoMut* is available on the GitHub repository at https://github.com/DEIB-GECO/YoyoMut (accessed on 7 April 2026); the web interface can be accessed at https://gmql.eu/yoyomut/ (accessed on 7 April 2026).

## Abbreviations

The following abbreviations are used in this manuscript:

API	Application Programming Interface
ARG	ARGinine amino acid
ORF	Open Reading Frame
PDB	Protein Data Bank
SARS-CoV-2	Severe Acute Respiratory Syndrome COronaVirus 2
